# Antimicrobial Resistance Gene Profiles in *Salmonella* spp., *Escherichia coli*, and *Enterococcus* spp. from Conventional and Antibiotic-Free Chicken Meat

**DOI:** 10.3390/pathogens15090899

**Published:** 2026-08-26

**Authors:** Camila Koutsodontis Cerqueira-Cézar, Evelyn Cristine da Silva, Aryele Nunes da Cruz Encide Sampaio, Evelyn Fernanda Flores Caron, Wanderson Sirley Reis Teixeira, Emanoelli Aparecida Rodrigues dos Santos, Larissa de Abreu Albano, Gustavo Guimarães Fernandes Viana, Larissa Soares de Araujo, João Pessoa Araújo Junior, Carlo Spanu, Fábio Sossai Possebon, Juliano Gonçalves Pereira

**Affiliations:** 1 Department of Animal Production and Preventive Veterinary Medicine, School of Veterinary Medicine and Animal Science, São Paulo State University (UNESP), Prof. Dr. Walter Mauricio Correa Street, Botucatu 18618-681, SP, Brazil; camila.cezar@unesp.br (C.K.C.-C.); aryele.sampaio@unesp.br (A.N.d.C.E.S.); evelyn.fernanda@unesp.br (E.F.F.C.); wanderson.teixeira@unesp.br (W.S.R.T.); emanoelli.santos@unesp.br (E.A.R.d.S.); larissa.albano@unesp.br (L.d.A.A.); ggf.viana@unesp.br (G.G.F.V.); soares.araujo@unesp.br (L.S.d.A.); fabio.possebon@unesp.br (F.S.P.); 2 Institute for Biotechnology, São Paulo State University (UNESP), Tecomarias Avenue, Botucatu 18607-440, SP, Brazil; evelyn.cristine@unesp.br (E.C.d.S.); joao.pessoa@unesp.br (J.P.A.J.); 3 Department of Veterinary Medicine, University of Sassari (UNISS), 07100 Sassari, Italy; cspanu@uniss.it

**Keywords:** antimicrobial resistance, poultry, foodborne pathogens, resistome, food safety, One Health

## Abstract

Antimicrobial resistance (AMR) is a global public health concern, and animal food production contributes to its spread. This study compared antimicrobial resistance gene (ARG) profiles in *Salmonella* spp., *Escherichia coli*, and *Enterococcus* spp. isolated from chicken meat produced under conventional (CONV) and antibiotic-free (ATB-Free) systems using whole-genome sequencing of 207 phenotypically selected isolates. We hypothesized that the ATB-Free production would be associated with fewer resistance genes, but the observed patterns differed among the bacterial groups. In *E. coli* (41 genes; 61.0% shared between chains), the gene load per isolate was similar between the production systems (median of four in both chains). In *Salmonella* spp., only five ATB-Free isolates were available, precluding a meaningful comparison between the production systems; a rarefaction analysis showed that the lower gene count observed in this group was consistent with the sampling effort. In *Enterococcus* spp., the CONV isolates carried a higher gene load (median of two vs. one) and a higher proportion of enzymatic determinants (48.4% vs. 11.8%). This difference was concentrated in isolates carrying the ionophore resistance genes *narA* and *narB* (CONV 41.9% vs. ATB-Free 5.9%; *p* < 0.001), which were not associated with transferable vancomycin or high-level aminoglycoside resistance determinants in this dataset. The resistance determinants associated with the clinically important antimicrobial classes, including *bla*_CTX-M-8_, quinolone resistance genes, and fosfomycin resistance genes, were detected in isolates from both production systems. Overall, the observed ARG profiles differed among the bacterial groups, indicating that the relationship between the production system and the resistome was not uniform. Since the sequenced isolates constituted a phenotypically selected subset, the observed ARG frequencies should not be interpreted as population-level prevalence estimates for either production system.

## 1. Introduction

Antimicrobial resistance (AMR), though a natural evolutionary process, is significantly intensified by human activities. The misuse and overuse of antimicrobials in clinical settings remain the primary drivers of this issue [1]. Additionally, the widespread use of antimicrobials in agriculture and livestock production accelerates the environmental dissemination of resistant strains [2]. These interconnected sources of resistance highlight the need for coordinated surveillance across human, animal, and environmental sectors [3]. Ongoing surveillance remains necessary to identify the emerging resistance patterns and evaluate the effectiveness of mitigation measures [4].

Despite the expanding scale of poultry production, the industry has achieved notable progress in reducing antimicrobial use (AMU) over the past decade. The data indicates that, while the global Population Correction Unit for chickens, a measure of the total biomass of the animals at risk of antimicrobial treatment, rose by 38% between 2010 and 2020, AMU in this sector actually decreased by 67%. This represents a 76% reduction in the average AMU per production unit, reflecting a significant shift towards more responsible and prudent antimicrobial practices [5]. Antibiotic-free (ATB-Free) farming methods have been promoted as a strategy to reduce AMU in livestock [6], thereby decreasing the selective pressure on bacteria and mitigating the development and spread of AMR, including resistance genes [7].

Notably, countries with high antimicrobial usage in food-producing animals, such as the United States, tend to have higher rates of AMR compared to countries with lower usage levels, such as Sweden and New Zealand [8]. This observation supports the association between antimicrobial use practices and the resistance patterns observed in animal populations [9]. The transmission of AMR from animals to humans can occur through multiple pathways, with the most prevalent being the direct oral route, which includes the consumption of contaminated meat and the ingestion of feces through tainted food or water. Additionally, direct contact between humans and animals represents another significant transmission route [10].

Comparative studies of conventional (CONV) and ATB-Free poultry have produced inconsistent results. In Italy, antimicrobial resistance gene (ARG) occurrence did not differ conclusively between farming systems, and the authors attributed the persistence of resistance to environmental reservoirs and previous antimicrobial use rather than to current practices [11]. A subsequent assessment of the Italian chain reported a lower ARG distribution in ATB-Free carcasses [12]. In the United States, a genomic characterization of *Salmonella* from no-antibiotics-ever and CONV broiler complexes identified differences for specific determinants rather than a uniform reduction [13]. In Brazil, a genomic comparison between the two systems has so far been restricted to extended-spectrum beta-lactamase (ESBL)-producing *Escherichia coli* recovered by selective screening [14]. The available evidence remains difficult to compare because the studies have focused on different bacterial species, resistance mechanisms, and sampling strategies, and no study has assessed whether the effect of ATB-Free production is consistent across bacterial groups with contrasting resistance repertoires.

The World Health Organization maintains a list of Medically Important Antimicrobials that identifies last-resort agents and those at greatest risk from rising resistance [15]. The determinants associated with these agents were of particular interest in the present analysis. We previously characterized the phenotypic resistance profiles of this isolate collection [16]. Here, we report the genomic characterization of part of the same collection, comparing the frequency and diversity of AMR genes in *Salmonella* spp., *E. coli*, and *Enterococcus* spp. recovered from chicken meat produced under CONV and ATB-Free systems. We hypothesized that ATB-Free production would be associated with a lower frequency and diversity of AMR genes. To our knowledge, this is the first study in Brazil to compare all three of these bacterial groups simultaneously by whole-genome sequencing (WGS) across the two production systems, allowing an assessment of whether any effect of the production system is consistent across Gram-negative and Gram-positive organisms.

## 2. Materials and Methods

### 2.1. Isolation and Selection of Bacterial Isolates for WGS

The collection and initial processing of poultry samples have been previously described [16]. Briefly, poultry cuts (*n* = 284) from both CONV and ATB-Free chains were collected in Botucatu, São Paulo, Brazil, and screened for *Salmonella* spp., *E. coli*, and *Enterococcus* spp. All isolates included in the present study had been previously molecularly confirmed by polymerase chain reaction (PCR), as described in [16]. For the ATB-Free group, certification of the antibiotic-free production system, as indicated on the commercial product packaging, was used as an inclusion criterion. Products without such certification were not included in this group.

A total of 592 isolates were recovered and tested for antimicrobial susceptibility using the disk diffusion method. From these, 207 isolates were selected by convenience for WGS, with the aim of including isolates exhibiting different antimicrobial susceptibility profiles observed in the original collection. A total of 189 (91.3%) were found to harbor at least one resistance gene. The final dataset comprised 59 *E. coli* isolates (36 ATB-Free; 23 CONV), 99 *Enterococcus* spp. isolates (68 ATB-Free; 31 CONV), and 31 *Salmonella* spp. isolates (5 ATB-Free; 26 CONV).

### 2.2. DNA Extraction

For DNA extraction, each microorganism followed a distinct process. For *Salmonella* spp. and *E. coli*, after incubating the inoculated culture in brain heart infusion (BHI), 400 µL was aliquoted into a 1.5 mL microcentrifuge tube, centrifuged at 4500 rpm for 5 min, and the supernatant was discarded. The pellet was washed in 400 µL of phosphate-buffered saline (PBS), centrifuged again, then resuspended in 200 µL of DNase-free water (Invitrogen, Carlsbad, CA, USA), incubated at 100 °C for 10 min, centrifuged, and the supernatant was stored at −20 °C [17,18]. *Enterococcus* spp. required a different approach: the pellet was washed three times in 500 µL of 1× Tris-EDTA (TE) buffer (New England Biolabs, Ipswich, MA, USA), then resuspended in 100 µL of 1× TE buffer, incubated at 98 °C, centrifuged at 12,000 rpm, and the supernatant stored at −20 °C [19]. Heat-based DNA extraction methods have also been successfully applied for bacterial whole-genome sequencing using Illumina platforms, supporting the applicability of this rapid approach for sequencing purposes [20].

### 2.3. Next-Generation Sequencing (NGS) and Bioinformatics Analysis

Quantification of the extracted DNA was performed using an Invitrogen Qubit 2.0 fluorometer (Thermo Fisher Scientific, Waltham, MA, USA) with a Qubit^®^ 1× dsDNA HS (High Sensitivity) Assay Kit (Invitrogen, Thermo Fisher Scientific, Waltham, MA, USA). For library preparation, 250 ng of DNA was used as input, and libraries were prepared using an Illumina DNA Prep Kit (Illumina Inc., San Diego, CA, USA) according to the manufacturer’s recommendations.

The prepared libraries were quantified using a Qubit 2.0 fluorometer, and fragment-size distribution was assessed by capillary electrophoresis using a 4150 TapeStation system (Agilent Technologies, Santa Clara, CA, USA) with High Sensitivity D1000 ScreenTape (Agilent Technologies). The libraries were sequenced on an Illumina MiSeq platform (Illumina Inc.) using a 600-cycle flow cell cartridge (Illumina Inc.), with a target sequencing depth of approximately 50× per isolate. DNA quantification and sequencing and assembly metrics for each isolate are provided in Supplementary Table S5.

Sequencing data obtained from Illumina platforms were subjected to quality control and processing steps. Initial quality assessment was performed using FastQC (v0.12.1) [21] to ensure the integrity of the sequencing data. Subsequently, Trimmomatic (v0.39) [22] was employed to trim low-quality reads and adapter sequences [23] to reconstruct the genomic sequences. Functional annotations were conducted with reference to the UniProtKB database [24] to identify and categorize gene functions. AMR genes were specifically identified through the ResFinder (v4.7.2) [25] pipeline, which provided detailed insights into resistance profiles based on sequence data.

### 2.4. Data Analysis

The frequency of resistance genes among the sequenced *Salmonella* spp., *E. coli*, and *Enterococcus* spp. isolates from CONV and ATB-Free poultry was compared using the chi-square test. Whenever ≥25% of expected counts were <5 (or any expected count <1), the two-sided Fisher’s exact test was applied. The same tests were applied to compare the proportion of isolates carrying at least one gene of each mechanism class (efflux, enzymatic, and target site) between chains. Effect sizes were expressed as odds ratios (ORs; CONV vs. ATB-Free) with 95% confidence intervals (CIs) from PROC FREQ in SAS 9.4 (SAS Institute Inc., Cary, NC, USA). *p*-values across genes were adjusted for multiple testing using the Benjamini–Hochberg false discovery rate (FDR) in PROC MULTTEST, yielding *q*-values. The significance threshold was α = 0.05. Continuous comparisons of gene load per isolate between production chains were performed using the Mann–Whitney *U* test, and comparisons of gene load across *Salmonella* serovars using the Kruskal–Wallis test. Gene load per isolate was additionally compared between *Enterococcus* spp. isolates grouped by *narAB* carriage, both within the conventional chain (*narAB*-positive vs. *narAB*-negative) and between conventional *narAB*-negative and antibiotic-free isolates, using the Mann–Whitney *U* test.

Co-occurrence between *narAB* and each of the other resistance genes detected in the conventional chain was tested using Fisher’s exact test on presence–absence data. This formed a separate family of 20 comparisons, corresponding to all resistance genes detected in the conventional chain except *narA* and *narB*, to which the Benjamini–Hochberg correction was applied independently of the between-chain prevalence analysis.

For *Salmonella* spp. isolates, additional analyses were performed, including in silico serovar determination using SeqSero2 (https://github.com/denglab/SeqSero2, accessed on 20 August 2025) and SerotypeFinder 2.0 (https://cge.food.dtu.dk/services/SerotypeFinder/, accessed on 20 August 2025).

To evaluate the association between *Salmonella* serovars and ARGs, a bipartite network analysis was performed separately for the ATB-Free and CONV systems. Networks were constructed with nodes representing serovars and ARGs, and edges linking a serovar to each ARG detected in it, weighted by the number of isolates in which the serovar–gene pairing occurred. Network topology was characterized by weighted degree (node strength), betweenness centrality, eigenvector centrality, and modularity class, and the relative importance of nodes was assessed by ranking their weighted degree. Nodes acting as bridges between different network modules were also identified. Analyses were performed in R (v. 4.4.2) using the igraph (v. 2.1.4), tidygraph (v. 1.3.1), and ggraph (v. 2.2.1) packages.

To assess whether the smaller number of resistance genes and network nodes observed in the ATB-Free chain could be explained by sampling effort, a permutation procedure was applied to the *Salmonella* data. Five CONV isolates were drawn at random without replacement, and the number of distinct resistance genes, network nodes, and network edges was recorded. This was repeated 10,000 times using PROC SURVEYSELECT in SAS 9.4. The values observed in the five ATB-Free isolates were then located within the resulting distributions, and the proportion of permutations yielding a value less than or equal to the observed ATB-Free value was reported. This resampling procedure applies the principle of rarefaction, which adjusts for differences in sampling effort when comparing richness between samples [26].

## 3. Results

Among the 207 isolates subjected to NGS, 189 (91.3%) harbored at least one AMR gene, comprising 110 of 117 (94.0%) isolates from the ATB-Free chain and 79 of 90 (87.8%) from the CONV chain (*p* = 0.14).

### 3.1. Antimicrobial Resistance Gene Profiles in E. coli Isolates

**Figure 1:**
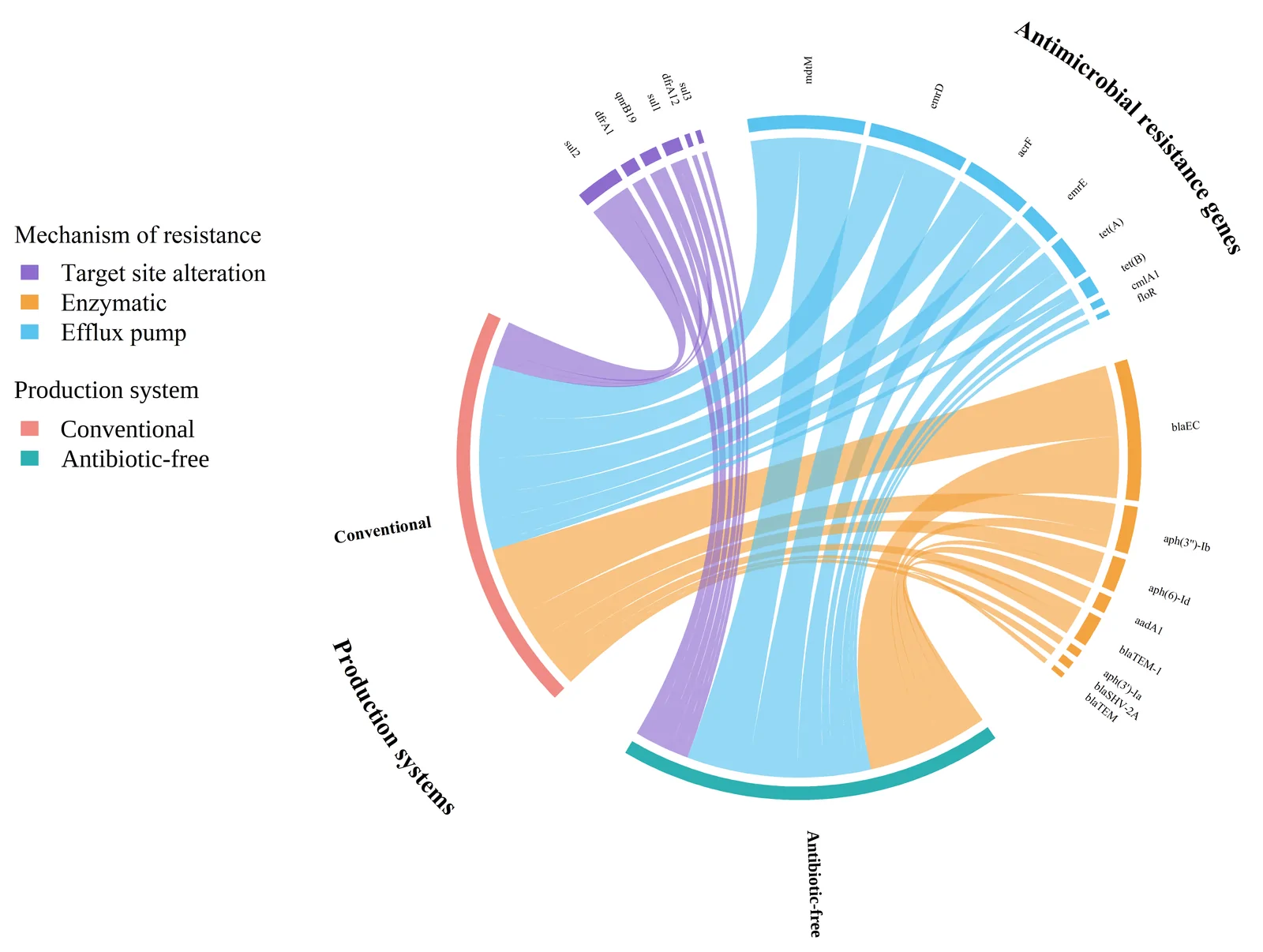
Chord diagram of the antimicrobial resistance genes detected in *Escherichia coli* isolates from chicken meat produced under conventional and antibiotic-free systems. Each ribbon links a production system to the resistance genes with highest frequencies detected in that system. Ribbon color indicates the mechanism of resistance, and ribbon width corresponds to the frequency of the gene within that system. Exact gene frequencies are given in the Supplementary Table S1.

A total of 41 resistance genes were identified in the *E. coli* isolates, representing the highest number among the three pathogens analyzed. Both of the sample sources shared 25 (61.0%) of the identified genes, with the three most prevalent genes being *bla*_EC_ (76.3%, ATB-Free 72.2% vs., CONV 82.6%; OR = 1.83, *p* = 0.53), *mdtM* (66.1%, ATB-Free 72.2% vs. CONV 56.5%; OR = 0.50, *p* = 0.27), and *emrD* (55.9%, ATB-Free 58.3% vs. CONV 52.2%; OR = 0.78, *p* = 0.79) (Figure 1). In terms of the mechanism per isolate, most carried at least one efflux gene (ATB-Free 91.7%, CONV 91.3%; OR = 0.95, *p* = 1.00) and enzymatic gene (ATB-Free 86.1%, CONV 95.7%, OR = 3.55, *p* = 0.39), whereas target site alteration occurred in 44.1% of the isolates (ATB-Free 38.9%, CONV 52.2%; OR = 1.71, *p* = 0.42). The gene load per isolate was similar between the chains (median ATB-Free = 4 [interquartile range (IQR) 3–6.25] vs. CONV = 4 [IQR 3–7]). The comprehensive results for all the genes are summarized in Table S1.

Figure 1 provides an integrated view of the resistance genes detected in *E. coli*, linking each gene to its production system and mechanism of resistance.

Additionally, the analysis revealed that 11 genes (26.8%) were exclusive to the isolates from the ATB-Free system, while only 5 genes (12.2%) were unique to the CONV system. In the ATB-Free samples, the exclusive genes comprised ESBL variants (*bla*_CTX-M-8_, *bla*_CTX-M-55_), *fos* determinants (*fosA*, *fosA3*), *bla*_ACT-90_, *bleO*, *lnu*(A) and *oqxA*/*oqxB*; these results suggest that the ATB-Free system harbors unique β-lactam and fosfomycin/quinolone-associated resistance genes absent in the CONV system. In the CONV chain meat cuts, the main resistance genes were *bla*_SHV-2A_, *aac(3)-IId*, *lnu*(G), *dfrA14* and *qnrS1*.

### 3.2. Antimicrobial Resistance Gene Profiles in Salmonella spp. Isolates

For the *Salmonella* spp. isolates subjected to NGS, a total of 20 resistance genes were identified. Notably, all 20 genes were present in the isolates from the CONV system, whereas only 8 (40.0%) of these genes were found in the ATB-Free isolates. This disparity is consistent with the smaller number of ATB-Free isolates (*n* = 5). When five CONV isolates were resampled at random (10,000 permutations), the median number of distinct genes recovered was 10, and the 8 genes observed in the ATB-Free chain fell within the central range of this distribution, being recovered in 29.5% of permutations. The lower gene diversity observed in the ATB-Free chain is therefore within the range expected from the sampling effort alone (Table S2). The gene load per isolate was similar between the chains (median 6 [IQR 5–6] in ATB-Free vs. 6 [IQR 2–7] in CONV; *p* = 0.74).

**Figure 2:**
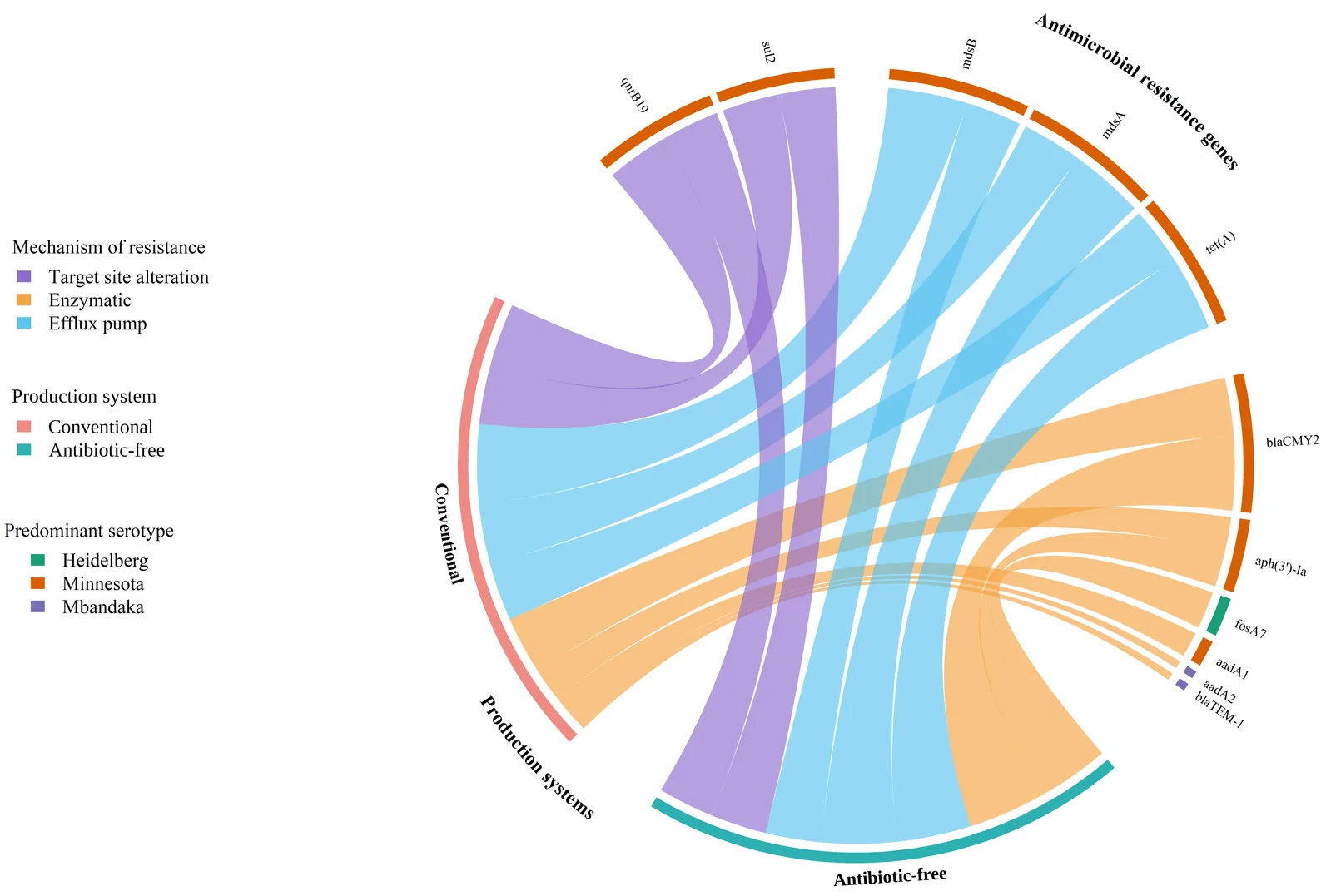
Chord diagram of the antimicrobial resistance genes detected in *Salmonella* spp. isolates from chicken meat produced under conventional and antibiotic-free systems. Each ribbon links a production system to the resistance genes with highest frequencies detected in that system. Ribbon color indicates the mechanism of resistance, and ribbon width corresponds to the frequency of the gene within that system. Colored markers on the gene arcs indicate the predominant *Salmonella* serovar associated with each gene. Only serovars that were predominant for at least one gene are shown. Exact gene frequencies are given in the Supplementary Table S2.

Among the sequenced *Salmonella* isolates, the six most frequently detected genes were *mdsB* (80.6%), *mdsA* (67.7%), *qnrB19* (67.7%), *bla*_CMY-2_ (64.5%), *tet*(A) (64.5%), and *sul2* (61.3%) (Figure 2). The comparison between the sequenced isolates from both production chains did not reveal statistically robust differences after a multiple-test correction (*q* = 1.00 for all genes), although a trend toward higher prevalence of *fosA7* in the ATB-Free system was observed (ATB-Free 40.0% vs. CONV 3.8%; OR = 0.06, *p* = 0.06). Genes such as *mdsB* (60.0% vs. 84.6%; OR = 3.67; *p* = 0.241) and *mdsA* (80.0% vs. 65.4%; OR = 0.47; *p* = 1.00) were frequent in the samples from both chains. Several determinants appeared only in the CONV isolates, such as *aadA1/2*, *bla*_TEM-1_, *bla*_CTX-M-8_, *catA*, *cmlA1*, *dfrA12*, *lnu*(F), *qnrS1*, *sul3*, *tet*(J) and *dfrA1*, while no gene was exclusive to the ATB-Free samples, although the detection of an exclusive gene is unlikely with only five isolates. In terms of mechanisms, most of the isolates from both systems carried efflux pump and target-site alteration genes, and a substantial fraction carried enzymatic genes, with no significant differences between the chains (*p* > 0.2).

Figure 2 provides an integrated view of the resistance genes detected in *Salmonella* spp., linking each gene to its production system, mechanism of resistance, and predominant serovar.

Additionally, four *Salmonella* serovars were identified in the poultry samples: *Salmonella* Minnesota, Senftenberg, Mbandaka, and Heidelberg. The serovar Mbandaka was exclusively detected in isolates from the CONV system, while the other serovars were present in both production systems. However, the absence of *Salmonella* Mbandaka in the ATB-Free isolates may be due to the limited number of isolates from this group in this study. Among the serovars, Minnesota was the most prevalent (19/31 isolates) and carried the highest number of resistance genes per isolate (median 7, IQR 5.5 to 8), compared with Heidelberg (median 6), Mbandaka (median 4.5), and Senftenberg (median 2) (Kruskal–Wallis, *p* = 0.004). The serovar and the production system were confounded in this study, since 17 of the 19 Minnesota isolates originated from the CONV chain, so the contribution of each factor cannot be separated.

**Figure 3:**
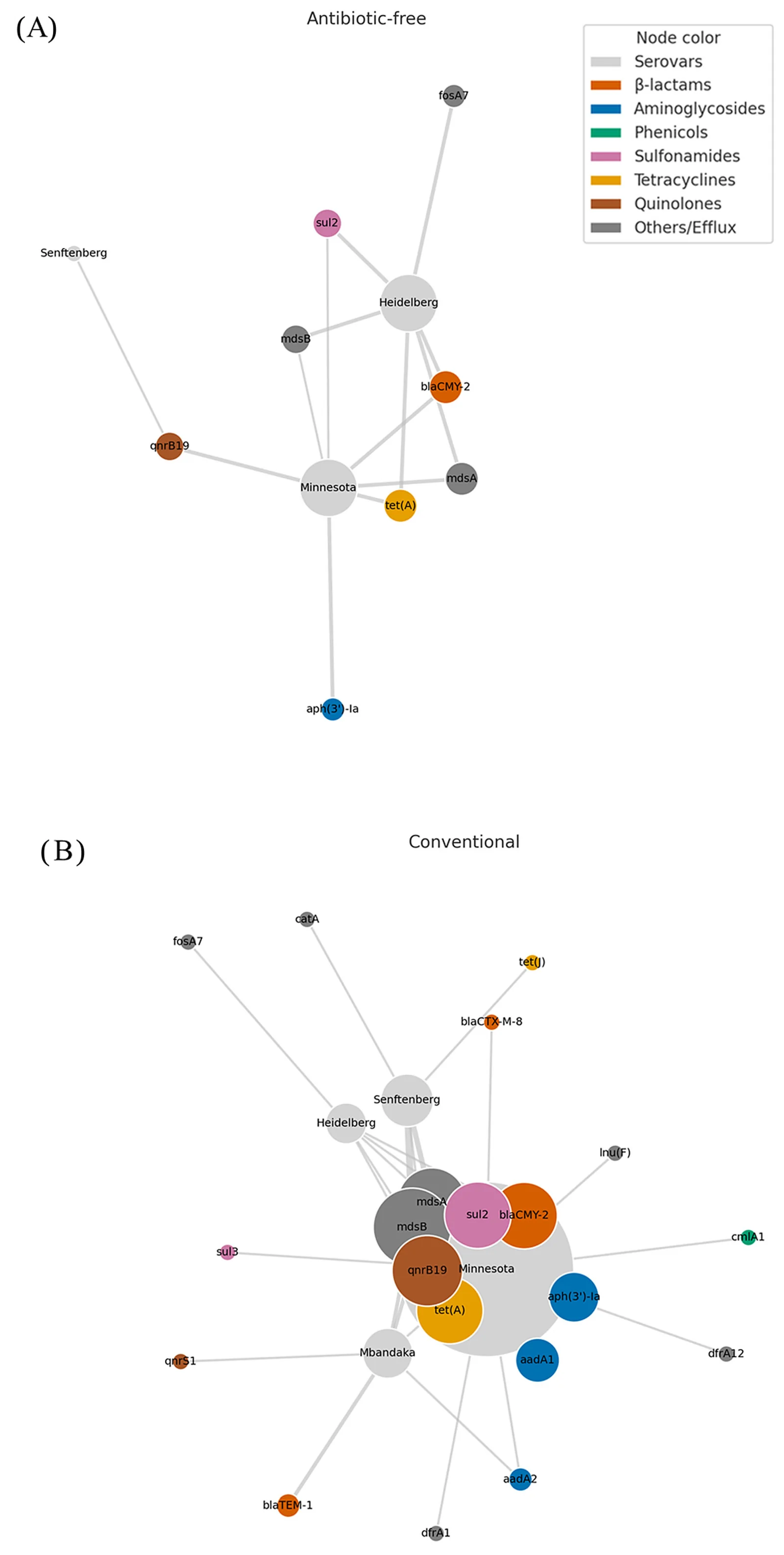
Serovar–antimicrobial resistance gene association networks for *Salmonella* spp. from chicken meat produced under antibiotic-free and conventional systems. Nodes represent *Salmonella* serovars (light gray) and antimicrobial resistance genes (colored by antimicrobial class). Node size is proportional to the total frequency of the serovar or gene; edges connect serovars to the genes detected in them, and edge thickness reflects the frequency of that gene within the serovar. (**A**) Antibiotic-free network, comprising 11 nodes (3 serovars, 8 genes) and 14 edges. (**B**) Conventional network, comprising 24 nodes (4 serovars, 20 genes) and 31 edges, with *S*. Minnesota as the principal hub. Networks were constructed separately for the two production systems. Because several gene nodes overlap the *S*. Minnesota node in (B), the exact node strengths and edge weights for both networks are listed in the Supplementary Table S3.

The network analysis described the structure of the serovar–gene associations within each system. The ATB-Free network comprised 11 nodes and 14 connections, while the CONV network contained 24 nodes and 31 connections. Since each node represents a serovar or gene detected in that system, the network size increases with the number of isolates sequenced (5 for ATB-Free and 26 for CONV). When five CONV isolates were resampled at random (10,000 permutations), the median network size was 12 nodes and 13 edges, and the 11 nodes and 14 edges observed in the ATB-Free network fell within these distributions (recovered in 42.0% and 63.8% of the permutations, respectively). The difference in the network size between the systems is therefore consistent with the sampling effort. Within the CONV network, *S*. Minnesota acted as the main hub, connected to 14 distinct genes with a weighted degree of 110, and the exclusive detection of *S*. Mbandaka, carrying genes such as *bla*_TEM-1_, *aadA2*, and *qnrS1*, expanded the diversity of the connections. *S*. Minnesota was linked to seven distinct genes in the ATB-Free network, reflecting the two Minnesota isolates available from that chain, and the number of genes per Minnesota isolate was similar in both systems (median 7 in CONV vs. 6 in ATB-Free) (Figure 3, Table S3).

### 3.3. Antimicrobial Resistance Gene Profiles in Enterococcus spp. Isolates

**Figure 4:**
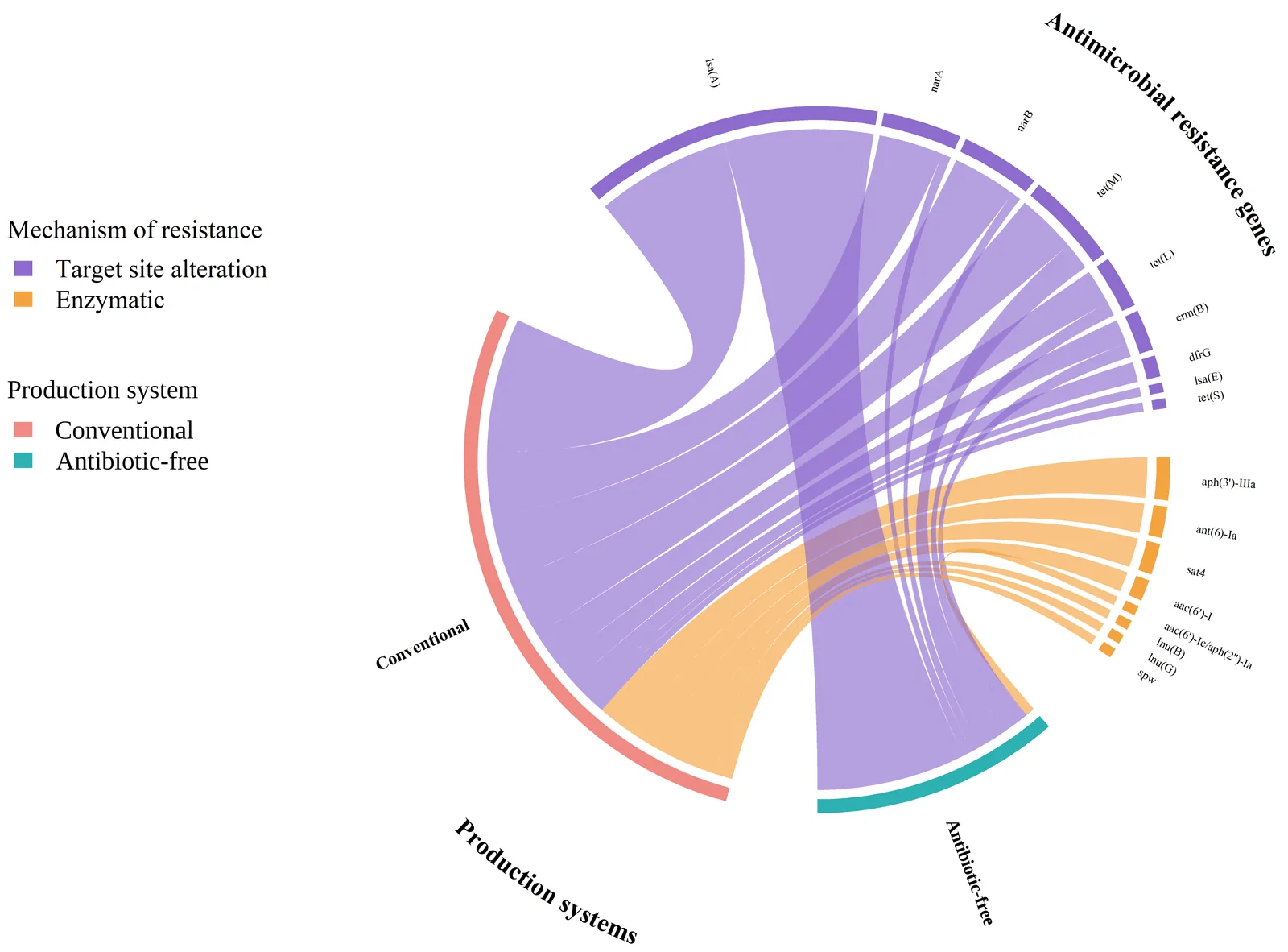
Chord diagram of the antimicrobial resistance genes detected in *Enterococcus* spp. isolates from chicken meat produced under conventional and antibiotic-free systems. Each ribbon links a production system to the resistance genes with highest frequencies detected in that system. Ribbon color indicates the mechanism of resistance, and ribbon width corresponds to the frequency of the gene within that system. Exact gene frequencies are given in the Supplementary Table S4.

Among the 99 *Enterococcus* spp. isolates derived from poultry cuts, a distinct resistance gene profile was observed compared to the previously analyzed Gram-negative isolates. A total of 28 resistance genes were identified. The CONV isolates exhibited 22/28 (78.6%) of these genes, while the ATB-Free samples presented 19/28 (67.9%) of the 28 genes; 13 genes were shared between both systems. Overall, the most frequently detected genes were *lsa*(A) (93.9%), *tet*(M) (24.2%), *narA* (17.2%) and *narB* (17.2%), *tet*(L) (13.1%), and *aph(3′)-IIIa* (11.1%) (Figure 4, Table S4).

The *lsa*(A) gene was the most frequently detected gene, detected in 93.9% of the isolates (ATB-Free 97.1%, CONV 87.1%). Regarding resistance mechanisms, almost all the isolates in this study carried at least one target-site alteration gene (ATB-Free 98.5%, CONV 93.5%), whereas enzymatic genes were more frequent in the CONV isolates (ATB-Free 11.8%, CONV 48.4%; *p* < 0.05). Consequently, the gene load per isolate (number of distinct genes) was higher in the CONV isolates (median 2, IQR 1–6) compared with the ATB-Free isolates (median 1, IQR 1–2). In the CONV samples, a higher frequency of *narB* and *narA* was observed, and they were detected in the same 17 isolates (ATB-Free 5.9% vs. CONV 41.9%; OR = 11.6, *p* < 0.001, *q* < 0.001).

A higher frequency of *tet*(M) was observed in the CONV chain samples (ATB-Free 17.6% vs. CONV 38.7%; OR = 3.0, *p* = 0.041, *q* = 0.144), whereas *tet*(L) showed lower and non-significant comparison frequencies across the chains (ATB-Free 8.8% vs. CONV 22.6%; OR 3.0, *p* = 0.104, *q* = 0.209). *tet*(S) and *tet*(O) were rare and the latter occurred only in meat from the ATB-Free chain.

Several aminoglycoside-associated genes were increased in the CONV samples, for example, *aph(3′)-IIIa* (ATB-Free 4.4% vs. CONV 25.8%; OR = 7.5, *p* = 0.004, *q* = 0.025) and *sat4* (ATB-Free 0.0% vs. CONV 19.4%, *p* = 0.001, *q* = 0.006), as well as *dfrG* (ATB-Free 0.0% vs. CONV 12.9%, *p* = 0.008, *q* = 0.047). The genes *ant(6)-Ia* (*p* = 0.025, *q* = 0.117) and *aac(6′)-I* (*p* = 0.032, *q* = 0.129) were also more frequent in the CONV samples, although only as a trend after FDR correction. Additionally, the *vanE*, *vanR_E_*, *vanS_E_*, *vanT_E_*, and *vanXY_E_* genes were detected together in a single ATB-Free isolate, which carried the complete *vanE* cluster, representing a descriptive finding.

Figure 4 provides an integrated view of the resistance genes detected in *Enterococcus* spp., linking each gene to its production system and mechanism of resistance.

### 3.4. Resistance Mechanisms from Genes Found in E. coli, Salmonella spp. and Enterococcus spp. and Their Comparisons Across Production Chains

The distribution of resistance mechanisms was broadly similar across the three bacterial groups, although some species-specific differences were observed (Table 1). When all the resistance genes from both production systems were pooled (*n* = 688), target-site alterations were the predominant mechanism, accounting for 40.7% of the resistance genes detected, followed by enzymatic inactivation (30.1%) and efflux pumps (29.2%). In *E. coli* (*n* = 286 detections), efflux pumps were the most frequent (46.5%), followed by enzymatic mechanisms (39.5%) and target-site alterations (14.0%). *Salmonella* spp. (*n* = 160) displayed a comparable distribution, with 42.5% of the genes related to efflux pumps, 30.0% to enzymatic mechanisms, and 27.5% to target-site alterations. In contrast, *Enterococcus* spp. (*n* = 242) presented a restricted profile, with no efflux-related genes and a clear predominance of target-site alterations (81.0%), while enzymatic determinants accounted for the remaining 19.0%. In terms of production systems, the relative proportions of mechanisms remained largely consistent between the CONV and ATB-Free isolates, suggesting that the production environment had little impact on the distribution of resistance pathways in our chicken meat samples.

## 4. Discussion

In our previous investigation of the phenotypic AMR in this isolate collection from chicken meat [16], we observed particularly concerning findings, including carbapenem resistance in *Salmonella* spp. and resistance to vancomycin and linezolid in *Enterococcus* spp. The prevalence of multidrug-resistance (MDR) varied across bacterial groups, with 90.7% of *Salmonella* spp. isolates classified as MDR, compared to 42.9% of *E. coli* and 12.0% of *Enterococcus* spp. isolates. The multiple antibiotic resistance (MAR) index further reflected these differences, averaging 0.35 for *Salmonella* spp., 0.12 for *E. coli*, and 0.09 for *Enterococcus* spp.

The frequency and diversity of AMR genes among the sequenced *Salmonella* spp., *E. coli*, and *Enterococcus* spp. isolates highlight the complexity of resistance patterns across bacterial groups and production systems. Notably, 91.3% of the sequenced isolates harbored at least one AMR gene. However, this high proportion should be interpreted in light of the phenotypic pre-selection of the isolates for WGS and should not be considered an estimate of ARG prevalence in the original bacterial population or in retail chicken meat. Livestock is a major source of acquired ARGs [27], particularly in low- and middle-income countries [28]. Although several studies have suggested a higher prevalence of pathogens and resistance are found in CONV rearing [8,29,30], our findings revealed the frequent detection of AMR genes among the sequenced isolates from both production systems. However, the effect of the production system was not uniform across the bacterial groups. In *E. coli*, where the sampling was comparable between the production chains (36 vs. 23 isolates), no gene differed after correction, and the gene load per isolate was equivalent. In *Salmonella* spp., the small number of ATB-Free isolates (*n* = 5) precluded a meaningful comparison. In *Enterococcus* spp., by contrast, the CONV isolates carried significantly more resistance genes per isolate and were enriched for several determinants.

As shown in Table 1, *E. coli* and *Salmonella* spp. display a predominance of resistance genes associated with efflux pumps, whereas *Enterococcus* spp. are characterized mainly by target-site modification. In this study, no efflux pump genes were detected in *Enterococcus* spp., although *tet*(L), which was recovered here, has been described elsewhere as encoding an efflux pump [31]. This finding is consistent with reports of a high frequency of mobile efflux genes among *Enterobacteriaceae* and their association with plasmids and integrons, which can promote horizontal dissemination in animal-production environments [32,33,34]. By contrast, *Enterococcus* spp. exhibit a different repertoire in which ribosomal protection mechanisms and target-site modifications predominate, while efflux genes are more restricted [35,36,37]. This pattern is consistent with the known differences in the resistance architecture of Gram-positive and Gram-negative bacteria, where RND-type efflux systems play a limited role in Gram-positive organisms and ABC transporters and ribosomal protection proteins contribute more substantially to resistance [38,39]. It should be noted that the gene annotation was performed using ResFinder, a tool that can under-represent intrinsic chromosomal determinants and thus may limit the detection of certain efflux pumps in *Enterococcus* [40,41]. Enterococci possess numerous intrinsic resistance mechanisms and readily acquire additional resistance determinants through horizontal gene transfer [42,43]. Tolerance to conditions such as elevated temperatures, high salinity, and disinfectant exposure [44,45] contributes to the persistence of enterococci on environmental surfaces, including those found in healthcare settings [46].

Considering the *Salmonella* spp. network analysis (Figure 3), due to only five *Salmonella* spp. isolates being recovered from the ATB-Free chain, the two networks could not be meaningfully compared. When the CONV chain was resampled at the same effort level, the ATB-Free network fell near the center of the resulting distributions for both the nodes and the edges, rather than in either tail, indicating that its smaller size relative to the full CONV network reflects the sampling effort rather than a structural difference between the chains. Within the CONV network, *S*. Minnesota acted as the main hub, connected to 14 distinct genes with a weighted degree of 110, compared with 10 for Senftenberg, 9 for Mbandaka, and 6 for Heidelberg. This position reflected both its predominance among the CONV isolates and its gene load per isolate. The presence of multiple resistance determinants within the same serovar may facilitate the co-selection and maintenance of these genes under antimicrobial pressure. However, the co-occurrence networks represent the genes detected within the same isolates and do not establish their physical linkage on the same plasmid, transposon, or other mobile genetic element. Moreover, because multilocus sequence typing (MLST) and detailed genomic context analyses were not performed, the observed co-occurrence patterns cannot distinguish between the physical linkage of the resistance determinants and their accumulation within clonally related isolates. Previous studies have linked the prominence of *S*. Minnesota to the presence of large plasmids carrying not only resistance but also virulence and heavy metal tolerance genes, which may enhance its capacity to persist and spread in poultry production environments [47]. Similarly, several surveys in Brazil have repeatedly reported *S*. Minnesota and *S*. Heidelberg as important carriers of diverse resistance genes, many of which were also detected in our dataset [48]. The evidence for plasmid-mediated transfer further supports this reservoir role, as conjugation experiments have demonstrated that *S.* Minnesota isolates harboring *bla*_CMY-2_ and *qnrB19* can transmit these genes, along with other linked determinants such as those conferring tetracycline and sulfonamide resistance, to *E. coli* recipients [49].

Our study identified a diverse range of AMR genes, including those associated with the Highest Priority Critically Important Antimicrobials as designated by the World Health Organization [15]. Among these, *oqxA*, *oqxB*, *qnrB19*, and *qnrS1* confer resistance to quinolones, a class valued for its broad-spectrum activity and oral availability that is used to treat urinary tract infections, gastroenteritis, community-acquired pneumonia, and infections of the skin and soft tissues [50]. The detection of *fosA*, *fosA3*, and *fosA7* is also relevant, since fosfomycin is used against infections caused by carbapenemase- and ESBL-producing *Enterobacterales*, methicillin-resistant *Staphylococcus aureus*, glycopeptide-resistant enterococci, and MDR *Pseudomonas aeruginosa* [51]. Although prohibited in veterinary medicine in Europe and China, fosfomycin continues to be used in South and Central America, including Brazil [52], which is consistent with the detection of these determinants in the isolates from Brazilian poultry.

Resistance to tetracyclines is frequently associated with MDR bacteria and can co-select for resistance to antibiotics of critical importance in human medicine [53]. Tetracyclines are among the antibiotic classes most heavily used in food-producing animals [54], and high tetracycline resistance has been widely reported in Brazilian poultry, including 94.0% of *Salmonella* spp. isolates from chicken meat in one national survey [55].

The genomic data did not account for several resistance phenotypes reported previously for this collection. Vancomycin resistance was recorded in 20.5% of the CONV and 23.5% of the ATB-Free *Enterococcus* spp. isolates, and linezolid resistance in 7.5% and 7.2%, respectively [16], whereas *van* genes were detected here in a single isolate and no acquired oxazolidinone resistance determinant was identified. The isolate carrying *van* genes harbored the complete *vanE* cluster, which is associated with low-level vancomycin resistance and is generally chromosomally encoded [56,57]. The phenotype–genotype discordance may arise because phenotypic susceptibility reflects both acquired and intrinsic mechanisms, whereas the genomic screening performed here primarily targeted known acquired resistance determinants. In addition, resistance may result from chromosomal mutations, altered gene expression, or intrinsic mechanisms that are not captured by acquired resistance-gene databases. The inclusion of isolates with intermediate susceptibility in the previous phenotypic classification may also have contributed to this discrepancy. Similarly, the imipenem resistance previously reported in 2.3% of CONV *Salmonella* spp. isolates was not accompanied by a detected carbapenemase gene, indicating that the observed phenotype cannot be attributed to an acquired carbapenemase based on the present genomic analysis.

Among all the determinants evaluated, *narA* and *narB* showed the strongest association with the production system (ATB-Free 5.9% vs. CONV 41.9%; OR = 11.6, *p* < 0.001, *q* < 0.001). These genes encode an ABC-type transporter conferring reduced susceptibility to ionophores, which are used as anticoccidial feed additives in poultry production [58]. However, farm-level records on antimicrobial and ionophore use were not available for the retail products analyzed in this study. Therefore, although the higher occurrence of *narA* and *narB* among the CONV isolates is compatible with the differences in selective pressures between the production systems, it cannot be directly attributed to ionophore exposure in the flocks from which the sampled products originated. Whether ionophore use carries indirect consequences for clinically relevant resistance is debated. A global analysis of 2442 *narAB*-bearing isolates from 51 countries reported that *narAB* was statistically associated with multiple resistance determinants, including the *vanA* cluster in *E. faecalis* [59]. A subsequent analysis of publicly available genomes reported that vancomycin resistance genes and the important aminoglycoside resistance gene *aac(6′)-aph(2″)* were absent from the 20 kb regions flanking *narAB* in broiler isolates [60]. This conclusion was challenged on the grounds that co-selection is driven by genetic linkage rather than by physical proximity, so a fixed distance threshold cannot exclude it [61]. In reply, the authors maintained that any such association must be demonstrated within the population experiencing ionophore selection rather than pooled across host species [62]. Among the CONV isolates in our study, those carrying *narAB* harbored more resistance genes than those without the genes (mean 7.0 vs. 1.7, *p* < 0.001), and *narAB* was associated with *sat4* and *aph(3′)-IIIa* (both *q* = 0.04), with a weaker association for *tet*(L) (*q* = 0.08). No association was observed with *aac(6′)-Ie/aph(2″)-Ia* (*q* = 0.56), and the *vanE* cluster was not detected in the CONV chain, being restricted to a single ATB-Free isolate that did not carry *narAB*. These observations are consistent with the co-selection of streptothricin and aminoglycoside determinants alongside *narAB* but provide no evidence of linkage to the determinants of greatest concern for human infection. Several limitations apply. Only 13 CONV isolates carried *narAB*. The species and sequence types were not determined, although the detection of *lsa*(A) in 93.9% of the isolates is consistent with a predominantly *E. faecalis* collection. Plasmid and mobile element analyses were not performed, and co-occurrence within a genome may reflect clonal structure rather than linkage on a transferable element.

The clearest difference between the chains was confined to *Enterococcus* spp., where the CONV isolates carried a higher gene load per isolate (median 2 vs. 1, *p* < 0.001) and a higher proportion of the CONV isolates carried at least one enzymatic determinant (48.4% vs. 11.8% of ATB-Free isolates). The gene load did not differ between the chains in *E. coli* (*p* = 0.72) or *Salmonella* spp. (*p* = 0.74). Within *Enterococcus* spp., these differences were concentrated in the *narAB*-carrying subpopulation. The CONV isolates that did not carry *narAB* had a similar gene load as the ATB-Free isolates (mean 1.67 vs. 1.78, *p* = 0.99), whereas the CONV isolates carrying *narAB* carried a mean of seven genes. The determinants enriched in the CONV chain, including *sat4*, *aph(3′)-IIIa*, and *dfrG*, are among those associated with *narAB* rather than reflecting a broad increase across the enterococcal population. By contrast, the determinants circulating in *E. coli* were equivalent between the chains, consistent with reports that resistance in this group is sustained not only by current on-farm administration but also by environmental reservoirs and prior use, which can maintain resistance even where antimicrobial use is reduced [63,64]. These observations suggest that withdrawing antimicrobials from poultry production may be detectable in the resistome where selection is intense and continuous but is not detectable in the broadly disseminated resistome.

This study has several limitations. First, the isolates submitted to WGS constituted a convenience subset of the original collection, selected to include different phenotypic antimicrobial susceptibility profiles rather than thorough random sampling. This selection strategy allowed the characterization of a diverse range of resistance determinants; however, it introduces selection bias and precludes interpreting the ARG frequencies in the sequenced subset as population-level prevalence estimates for bacteria recovered from either production system. Accordingly, the comparisons between the CONV and ATB-Free isolates should be interpreted as comparisons within this selected collection and should not be extrapolated to the overall bacterial populations of the two production systems. Second, only five *Salmonella* spp. isolates were recovered from the ATB-Free chain, which precluded a meaningful comparison between the production systems for this genus and limited the interpretation of the gene diversity and the network structure. Third, the gene annotation relied on ResFinder, which primarily identifies acquired determinants and under-represents intrinsic chromosomal mechanisms. This is particularly relevant for *Enterococcus* spp., in which no efflux determinants were detected, and the absence of detected efflux determinants should therefore not be interpreted as evidence for the absence of efflux-mediated resistance mechanisms. Fourth, although the *Enterococcus* isolates were molecularly confirmed at the genus level, WGS-based species identification and MLST were not performed, limiting the species-specific interpretation and preventing assessment of the contribution of clonal expansion to the observed resistance patterns. Fifth, detailed analyses of plasmid, transposon, and other mobile genetic element contexts were not performed. Therefore, gene co-occurrence within the isolates should not be interpreted as evidence for physical linkage or horizontal co-transfer. Finally, the isolates were recovered from retail chicken cuts collected in a single region over a limited period. Although the certification of the antibiotic-free production system was an inclusion criterion for the ATB-Free group, primary farm antimicrobial-use records were not available for independent verification by the researchers.

Longitudinal studies following the same production systems over time would help clarify whether changes in management practices influence resistance gene persistence. Including environmental samples from farms may help identify potential reservoirs contributing to the maintenance of resistance genes within production systems. These data would improve our understanding of how resistance circulates between farm environments and poultry products.

## 5. Conclusions

This study provides the first genomic comparison in Brazil of AMR genes in *Salmonella* spp., *E. coli*, and *Enterococcus* spp. from chicken meat produced under CONV and ATB-Free systems, examined across all three groups simultaneously.

Resistance determinants were detected in the isolates from both production systems, including genes associated with antimicrobial classes of clinical importance, such as *bla*_CTX-M-8_, quinolone resistance determinants, and the fosfomycin resistance genes *fosA*, *fosA3*, and *fosA7*. Their detection in the bacteria recovered from retail chicken meat highlights the presence of clinically relevant resistance determinants in the poultry production chain, although the present genomic analysis does not establish their expression, transferability, transmission to consumers, or their associated health risk. We hypothesized that ATB-Free production would carry a lower prevalence and diversity of AMR genes, but this depended on the bacterial group. The resistance was equivalent in *E. coli*, could not be assessed in *Salmonella* spp. because only five ATB-Free isolates were available, and diverged only in *Enterococcus* spp., where the CONV isolates carried a higher gene load concentrated in those bearing the ionophore resistance genes *narA* and *narB*. The determinants distinguishing the CONV chain were not the agents of greatest concern for human infection and showed no linkage to transferable vancomycin or high-level aminoglycoside resistance. These findings show that the effect of ATB-Free production on the resistome is not uniform and that a lower AMR burden cannot be assumed from the production label alone. These findings indicate that comparisons restricted to a single bacterial group may overlook important differences between production systems. Assessing multiple taxa simultaneously provides a broader view of resistance dissemination within poultry production.

## Figures and Tables

**Table 1:** Distribution of resistance mechanisms among the antimicrobial resistance genes detected in *Escherichia coli*, *Salmonella* spp., and *Enterococcus* spp. isolates from chicken meat produced under conventional and antibiotic-free systems. Values express the percentage of all detected resistance gene within each microorganism and production system that were assigned to each mechanism class. Gene detections correspond to isolate–gene pairs, so a gene detected in several isolates contributes once per isolate. These values are gene-based and are not directly comparable with the isolate-based percentages reported in the text.

Microorganism	Production System	Gene Detections (*n*)	Mechanism		
			Target Site Alteration	Efflux Pump	Enzymatic
*E. coli*	Antibiotic-free	168	13.7%	48.2%	38.1%
	Conventional	118	14.4%	44.1%	41.5%
*Salmonella* spp.	Antibiotic-free	25	24.0%	44.0%	32.0%
	Conventional	135	28.1%	42.2%	29.6%
*Enterococcus* spp.	Antibiotic-free	121	90.1%	0.0%	9.9%
	Conventional	121	71.9%	0.0%	28.1%

## Data Availability

The original contributions presented in this study are included in the article/Supplementary Materials. Further inquiries can be directed to the corresponding author.

## Supplementary Materials

The following supporting information can be downloaded at: https://www.mdpi.com/article/10.3390/pathogens15090899/s1, Table S1: Prevalence and odds ratios of antimicrobial resistance genes in *Escherichia coli* isolates from antibiotic-free and conventional poultry production systems; Table S2: Prevalence and odds ratios of antimicrobial resistance genes in *Salmonella* spp. isolates from antibiotic-free and conventional poultry production systems; Table S3: Network parameters of the serovar–gene association networks for *Salmonella* spp. from conventional and antibiotic-free chicken meat; Table S4: Prevalence and odds ratios of antimicrobial resistance genes in *Enterococcus* spp. isolates from antibiotic-free and conventional poultry production systems; Table S5: DNA quantification, sequencing, and genome assembly metrics for the 207 bacterial isolates subjected to whole-genome sequencing.
