# Supplementary material for: Antimicrobial Resistance Gene Profiles in Salmonella spp., Escherichia coli, and Enterococcus spp. from Conventional and Antibiotic-Free Chicken Meat
Source: Pathogens. 2026 Aug 26;15(9):899. doi: 10.3390/pathogens15090899 (PMC13610325; doi:10.3390/pathogens15090899)
Supplement: Supplementary file 1 [file pathogens-15-00899-s001.zip › pathogens-4502050-supplementary.pdf]

Supplementary Table S1. Prevalence and odds ratios of antimicrobial resistance genes in *Escherichia coli* isolates from antibiotic-free and conventional poultry production systems.

Gene	Mechanism	ATB-Free %	CONV %	OR	LCI	UCI	p	q
emrE	Efflux Pump	11.1	30.4	3.5	0.89	13.74	0.089	1.00
sul2	Target Site Alteration	13.9	34.8	3.31	0.92	11.85	0.105	1.00
aph(3'')-Ib	Enzymatic	16.7	34.8	2.67	0.78	9.09	0.129	1.00
aadA1	Enzymatic	19.4	4.3	0.19	0.02	1.64	0.133	1.00
bla_{SHV-2A}	Enzymatic	0.0	8.7	8.49	0.39	185.21	0.148	1.00
aph(6)-Id	Enzymatic	11.1	26.1	2.82	0.7	11.4	0.166	1.00
dfrA1	Target Site Alteration	16.7	4.3	0.23	0.03	2.03	0.229	1.00
mdtM	Efflux Pump	72.2	56.5	0.5	0.17	1.5	0.265	1.00
aac(3)-IId	Enzymatic	0.0	4.3	4.87	0.19	124.7	0.390	1.00
dfrA14	Target Site Alteration	0.0	4.3	4.87	0.19	124.7	0.390	1.00
lnu(G)	Enzymatic	0.0	4.3	4.87	0.19	124.7	0.390	1.00
qnrS1	Target Site Alteration	0.0	4.3	4.87	0.19	124.7	0.390	1.00
acrF	Efflux Pump	30.6	43.5	1.75	0.59	5.19	0.405	1.00
blaEC	Enzymatic	72.2	82.6	1.83	0.5	6.72	0.532	1.00
aph(3')-Ia	Enzymatic	2.8	8.7	3.33	0.28	39.04	0.554	1.00
tet(A)	Efflux Pump	19.4	26.1	1.46	0.42	5.07	0.748	1.00
emrD	Efflux Pump	58.3	52.2	0.78	0.27	2.23	0.789	1.00
aac(3)-IVa	Enzymatic	2.8	4.3	1.59	0.09	26.76	1.00	1.00
aac(3)-VIa	Enzymatic	2.8	4.3	1.59	0.09	26.76	1.00	1.00
aadA2	Enzymatic	2.8	4.3	1.59	0.09	26.76	1.00	1.00
aph(4)-Ia	Enzymatic	2.8	4.3	1.59	0.09	26.76	1.00	1.00
bla_{ACT-90}	Enzymatic	2.8	0.0	0.5	0.02	12.89	1.00	1.00
$bla_{CTX-M-55}$	Enzymatic	2.8	0.0	0.5	0.02	12.89	1.00	1.00
$bla_{CTX-M-8}$	Enzymatic	2.8	0.0	0.5	0.02	12.89	1.00	1.00
bla_{TEM}	Enzymatic	5.6	4.3	0.77	0.07	9.04	1.00	1.00
bla_{TEM-1}	Enzymatic	16.7	17.4	1.05	0.26	4.22	1.00	1.00
bleO	Enzymatic	2.8	0.0	0.5	0.02	12.89	1.00	1.00
cmlA1	Efflux Pump	8.3	4.3	0.5	0.05	5.12	1.00	1.00
dfrA12	Target Site Alteration	5.6	4.3	0.77	0.07	9.04	1.00	1.00
emhC	Efflux Pump	2.8	0.0	0.5	0.02	12.89	1.00	1.00
floR	Efflux Pump	5.6	4.3	0.77	0.07	9.04	1.00	1.00
fosA	Enzymatic	2.8	0.0	0.5	0.02	12.89	1.00	1.00
fosA3	Enzymatic	2.8	0.0	0.5	0.02	12.89	1.00	1.00
lnu(A)	Enzymatic	2.8	0.0	0.5	0.02	12.89	1.00	1.00
oqxA	Efflux Pump	2.8	0.0	0.5	0.02	12.89	1.00	1.00
oqxB	Efflux Pump	2.8	0.0	0.5	0.02	12.89	1.00	1.00
qnrB19	Target Site Alteration	11.1	8.7	0.76	0.13	4.54	1.00	1.00
sat2	Enzymatic	2.8	0.0	0.5	0.02	12.89	1.00	1.00
sul1	Target Site Alteration	11.1	8.7	0.76	0.13	4.54	1.00	1.00
sul3	Target Site Alteration	5.6	4.3	0.77	0.07	9.04	1.00	1.00
tet(B)	Efflux Pump	11.1	8.7	0.76	0.13	4.54	1.00	1.00

ATB-Free, antibiotic-free chain. CONV, conventional chain. OR, odds ratio for gene detection in conventional relative to antibiotic-free isolates. LCI and UCI, lower and upper limits of the 95% confidence interval. p, two-sided Fisher exact p-value. q, Benjamini-Hochberg false discovery rate adjusted p-value. Significance threshold alpha = 0.05. A 0.5 continuity correction was applied to genes with a zero cell.

Supplementary Table S2. Prevalence and odds ratios of antimicrobial resistance genes in *Salmonella* spp. isolates from antibiotic-free and conventional poultry production systems.

Gene	Mechanism	ATB-Free %	CONV %	OR	LCI	UCI	p	q
fosA7	Enzymatic	40.0	3.8	0.06	0.008157	0.83	0.06	1.00
mdsB	Efflux Pump	60.0	84.6	3.67	0.46	29.42	0.241	1.00
aadA1	Enzymatic	0.0	26.9	4.23	0.21	86.27	0.562	1.00
*bla*CMY-2	Enzymatic	80.0	61.5	0.40	0.04	4.11	0.631	1.00
tet(A)	Efflux Pump	80.0	61.5	0.40	0.04	4.11	0.631	1.00
aadA2	Enzymatic	0.0	7.7	1.12	0.05	26.83	1.00	1.00
*bla*TEM-1	Enzymatic	0.0	7.7	1.12	0.05	26.83	1.00	1.00
qnrB19	Target Site Alteration	60.0	69.2	1.50	0.21	10.79	1.00	1.00
mdsA	Efflux Pump	80.0	65.4	0.47	0.05	4.88	1.00	1.00
sul2	Target Site Alteration	60.0	61.5	1.07	0.15	7.54	1.00	1.00
sul3	Target Site Alteration	0.0	3.8	0.65	0.02	18.09	1.00	1.00
qnrS1	Target Site Alteration	0.0	3.8	0.65	0.02	18.09	1.00	1.00
dfrA12	Target Site Alteration	0.0	3.8	0.65	0.02	18.09	1.00	1.00
dfrA1	Target Site Alteration	0.0	3.8	0.65	0.02	18.09	1.00	1.00
cmlA1	Efflux Pump	0.0	3.8	0.65	0.02	18.09	1.00	1.00
catA	Enzymatic	0.0	3.8	0.65	0.02	18.09	1.00	1.00
*bla*CTX-M-8	Enzymatic	0.0	3.8	0.65	0.02	18.09	1.00	1.00
lnu(F)	Enzymatic	0.0	3.8	0.65	0.02	18.09	1.00	1.00
tet(J)	Efflux Pump	0.0	3.8	0.65	0.02	18.09	1.00	1.00
aph(3')-Ia	Enzymatic	40.0	34.6	0.79	0.11	5.66	1.00	1.00

ATB-Free, antibiotic-free chain. CONV, conventional chain. OR, odds ratio for gene detection in conventional relative to antibiotic-free isolates. LCI and UCI, lower and upper limits of the 95% confidence interval. p, two-sided Fisher exact p-value. q, Benjamini-Hochberg false discovery rate adjusted p-value. Significance threshold alpha = 0.05. A 0.5 continuity correction was applied to genes with a zero cell. Because the ATB-Free group comprised only 5 isolates, genes detected exclusively in the CONV chain (*catA*, *sul3*, *qnrS1*, *dfrA1*, *dfrA12*, *bla*CTX-M-8, *lnu*(F), and *tet*(J)) can yield an OR below 1 even though the gene was absent from the antibiotic-free chain. These estimates are unstable and should be read together with the prevalence values rather than on their own.

Supplementary Table S3. Network parameters of the serovar-gene association networks for *Salmonella* spp. from conventional and antibiotic-free chicken meat.

System	Node	Node type	Overlap	Bridge	Degree rank	Weighted degree (strength)	Betweenness	Eig.	Mod.
ATB-Free	*bla*CMY-2	gene	Yes	Yes	3	4	0	0.320	1
ATB-Free	*mdsA*	gene	Yes	Yes	4	4	0	0.320	2
ATB-Free	*tet*(A)	gene	Yes	Yes	5	4	0	0.320	1
ATB-Free	*mdsB*	gene	Yes	Yes	6	3	0.089	0.242	2
ATB-Free	*sul2*	gene	Yes	Yes	7	3	0.089	0.242	2
ATB-Free	*qnrB19*	gene	Yes	No	8	3	0.2	0.159	1
ATB-Free	*fosA7*	gene	Yes	No	9	2	0	0.165	2
ATB-Free	*aph(3')-Ia*	gene	Yes	No	10	2	0	0.155	1
ATB-Free	Heidelberg	serovar	Yes	Yes	1	12	0.233	0.514	2
ATB-Free	Minnesota	serovar	Yes	Yes	2	12	0.7	0.485	1
ATB-Free	Senftenberg	serovar	Yes	No	11	1	0	0.025	1
CONV	*mdsB*	gene	Yes	Yes	2	22	0.128	0.284	2
CONV	*qnrB19*	gene	Yes	Yes	3	18	0	0.314	1
CONV	*mdsA*	gene	Yes	Yes	4	17	0.413	0.209	2
CONV	*bla*CMY-2	gene	Yes	Yes	5	16	0	0.277	1
CONV	*sul2*	gene	Yes	Yes	6	16	0	0.277	1
CONV	*tet(A)*	gene	Yes	Yes	7	16	0	0.277	1

CONV	*aph(3′)-Ia*	gene	Yes	No	10	9	0	0.166	1
CONV	*aadA1*	gene	No	No	11	7	0	0.129	1
CONV	*aadA2*	gene	No	Yes	13	2	0.474	0.019	2
CONV	*bla*$_{TEM-1}$	gene	No	No	14	2	0	0.001	2
CONV	*fosA7*	gene	Yes	No	15	1	0	0.001	2
CONV	*lnu*(F)	gene	No	No	16	1	0	0.001	2
CONV	*qnrS1*	gene	No	No	17	1	0	0.001	2
CONV	*bla*$_{CTX-M-8}$	gene	No	No	18	1	0	0.018	1
CONV	*cmlA1*	gene	No	No	19	1	0	0.018	1
CONV	*dfrA1*	gene	No	No	20	1	0	0.018	1
CONV	*dfrA12*	gene	No	No	21	1	0	0.018	1
CONV	*sul3*	gene	No	No	22	1	0	0.018	1
CONV	*catA*	gene	No	No	23	1	0	0.001	2
CONV	*tet*(J)	gene	No	No	24	1	0	0.001	2
CONV	Minnesota	serovar	Yes	Yes	1	110	0.526	0.704	1
CONV	Senftenberg	serovar	Yes	Yes	8	10	0.249	0.054	2
CONV	Mbandaka	serovar	No	No	9	9	0.640	0.026	2
CONV	Heidelberg	serovar	Yes	Yes	12	6	0.328	0.035	2

ATB-Free, antibiotic-free chain. CONV, conventional chain. Overlap, node present in both networks. Bridge, node connecting otherwise separate modules or not. Degree rank, rank of the node by degree within its network. Degree, weighted degree (strength), that is, the sum of edge weights, where each edge weight is the number of isolates linking a serovar to a gene. Betweenness, betweenness centrality. Eig., eigenvector centrality. Mod., modularity class.

Supplementary Table S4. Prevalence and odds ratios of antimicrobial resistance genes in *Enterococcus* spp. isolates from antibiotic-free and conventional poultry production systems.

Gene	Mechanism	AF_%	CONV_%	OR	LCI	UCI	p	q
aac(6′)-I	Enzymatic	1.5	12.9	9.93	1.06	92.91	0.032	0.129
aac(6′)-Ie/aph(2′′)-Ia	Enzymatic	5.9	3.2	0.53	0.06	4.98	1.00	1.00
ant(6)-Ia	Enzymatic	4.4	19.4	5.2	1.21	22.41	0.025	0.117
aph(3′)-IIIa	Enzymatic	4.4	25.8	7.54	1.84	30.85	0.004	0.025
catA	Enzymatic	0.0	3.2	6.74	0.27	170.15	0.313	0.487
dfrG	Target Site Alteration	0.0	12.9	22.42	1.17	430.51	0.008	0.047
erm(B)	Target Site Alteration	8.8	16.1	1.99	0.56	7.09	0.313	0.487
lnu(B)	Enzymatic	0.0	6.5	11.61	0.54	249.34	0.096	0.206
lnu(C)	Enzymatic	1.5	3.2	2.23	0.14	36.91	0.53	0.733
lnu(G)	Enzymatic	0.0	6.5	11.61	0.54	249.34	0.096	0.206
lsa(A)	Target Site Alteration	97.1	87.1	0.2	0.04	1.18	0.075	0.206
lsa(D)	Target Site Alteration	1.5	3.2	2.23	0.14	36.91	0.53	0.733
lsa(E)	Target Site Alteration	0.0	6.5	11.61	0.54	249.34	0.096	0.206
msr(C)	Target Site Alteration	0.0	3.2	6.74	0.27	170.15	0.313	0.487
narA	Target Site Alteration	5.9	41.9	11.56	3.36	39.8	< 0.001	< 0.001
narB	Target Site Alteration	5.9	41.9	11.56	3.36	39.8	< 0.001	< 0.001
sat4	Enzymatic	0.0	19.4	34.92	1.9	642.46	0.001	0.006
spw	Enzymatic	0.0	6.5	11.61	0.54	249.34	0.096	0.206
tet(L)	Target Site Alteration	8.8	22.6	3.01	0.92	9.89	0.104	0.209
tet(M)	Target Site Alteration	17.7	38.7	2.95	1.13	7.66	0.041	0.144
tet(O)	Target Site Alteration	4.4	0.0	0.3	0.01	5.93	0.55	0.733
tet(S)	Target Site Alteration	2.9	6.5	2.28	0.31	16.95	0.587	0.747
vanE	Target Site Alteration	1.5	0.0	0.71	0.03	18.03	1.00	1.00
vanR$_E$	Target Site Alteration	1.5	0.0	0.71	0.03	18.03	1.00	1.00
vanS$_E$	Target Site Alteration	1.5	0.0	0.71	0.03	18.03	1.00	1.00
vanT$_E$	Target Site Alteration	1.5	0.0	0.71	0.03	18.03	1.00	1.00
vanXY$_E$	Target Site Alteration	1.5	0.0	0.71	0.03	18.03	1.00	1.00
vat(E)	Enzymatic	0.0	3.2	6.74	0.27	170.15	0.313	0.487

ATB-Free, antibiotic-free chain. CONV, conventional chain. OR, odds ratio for gene detection in conventional relative to antibiotic-free isolates. LCI and UCI, lower and upper limits of the 95% confidence interval. p, two-sided Fisher exact p-value. q, Benjamini-Hochberg false discovery rate adjusted p-value. Significance threshold alpha = 0.05. A 0.5 continuity correction was applied to genes with a zero cell.

Supplementary Table S5. DNA quantification, sequencing, and genome assembly metrics for the 207 bacterial isolates subjected to whole-genome sequencing.

ID Sample	Microorganism	Qubit dsDNA concentration (ng/µL)	Total DNA input (ng)	Quality-filtered reads	Mean sequencing coverage (×)	Genome Assembly size (bp)	Number of contigs	N50 (bp)	GC content (%)
1	*Escherichia coli*	6.84	250	490212	9.00	2977794	2911	1112	50.94
2	*Escherichia coli*	13.06	250	507064	9.00	3268597	2993	1213	50.95
3	*Escherichia coli*	26.50	250	1016592	22.00	4776132	1236	6173	50.42
12	*Escherichia coli*	10.20	250	490120	10.00	3395591	2841	1376	51.00
16	*Escherichia coli*	12.18	250	824474	19.00	4647765	1378	5935	50.84
17	*Escherichia coli*	9.92	250	582116	12.00	3604371	3029	1350	50.66
23	*Escherichia coli*	7.35	250	417360	7.00	2502819	2781	942	52.20
25	*Escherichia coli*	8.47	250	516212	11.00	3621239	2730	1576	50.90
26	*Escherichia coli*	11.68	250	486988	9.00	3312096	2871	1281	51.30
31	*Escherichia coli*	40.30	250	993894	19.00	5390255	1561	5443	50.39
44	*Escherichia coli*	7.32	250	2589096	47.00	5081540	264	40754	50.64
48	*Escherichia coli*	39.10	250	866400	18.00	4642075	1212	6115	50.53
52	*Escherichia coli*	39.00	250	475896	11.00	4006742	2728	1818	50.87
53	*Escherichia coli*	32.30	250	836584	19.00	4772792	1177	6687	50.63
54	*Escherichia coli*	6.64	250	634588	13.00	3676300	2979	1413	50.85
62	*Escherichia coli*	7.74	250	338876	6.00	2705520	2958	946	56.17
63	*Escherichia coli*	12.56	250	568242	11.00	3683941	3021	1415	51.07
67	*Escherichia coli*	25.00	250	1007100	21.00	5192807	1028	8151	50.38
71	*Escherichia coli*	31.30	250	319762	6.00	2643563	2799	987	50.83
73	*Escherichia coli*	43.90	250	785136	12.00	3833282	2774	1651	50.36
105	*Escherichia coli*	8.24	250	655084	12.00	4129142	3091	1622	51.01
106	*Escherichia coli*	8.00	250	360670	9.00	2847046	2771	1107	52.00
109	*Escherichia coli*	7.09	250	1619680	17.00	4767537	2250	2997	50.71
112	*Escherichia coli*	8.13	250	3024330	53.00	5504621	260	94583	50.44
114	*Escherichia coli*	5.92	250	3548410	82.00	4745017	97	89109	50.70
123	*Escherichia coli*	39.40	250	680998	12.00	3681668	3046	1382	50.06
129	*Escherichia coli*	6.32	250	532902	10.00	3163292	2901	1195	51.52
138	*Escherichia coli*	7.46	250	725574	18.00	4566513	1214	6634	50.71
144	*Escherichia coli*	11.61	250	3524998	74.00	4858660	171	68527	50.54
145	*Escherichia coli*	8.38	250	2558260	48.00	4949611	183	61147	50.44

155	*Escherichia coli*	40.10	250	802838	15.00	4575899	2045	3117	50.41
157	*Escherichia coli*	39.90	250	724502	14.00	4053941	2348	2215	50.66
159	*Escherichia coli*	7.27	250	1356278	21.00	4657750	758	10264	50.77
180	*Escherichia coli*	14.18	250	1387274	16.00	4645035	3101	1887	50.48
184	*Escherichia coli*	40.00	250	621264	12.00	3588513	3027	1356	50.32
193	*Escherichia coli*	38.80	250	743918	11.00	3531355	2870	1399	50.45
207	*Escherichia coli*	6.76	250	2079640	31.00	4756061	762	10580	50.61
209	*Escherichia coli*	7.89	250	1679230	26.00	5148338	574	17611	50.68
212	*Escherichia coli*	14.11	250	512008	11.00	3971010	2744	1760	51.09
219	*Escherichia coli*	9.04	250	2107258	36.00	4895970	298	30838	50.59
221	*Escherichia coli*	40.20	250	685276	13.00	3223218	2812	1275	50.29
228	*Escherichia coli*	36.30	250	912946	18.00	4951567	1897	3783	50.53
229	*Escherichia coli*	39.10	250	969516	13.00	6748650	4832	1701	46.16
231	*Escherichia coli*	43.90	250	460162	9.00	2738046	2776	1043	50.38
232	*Escherichia coli*	7.48	250	2629884	41.00	4746545	423	21307	50.63
237	*Escherichia coli*	13.67	250	2048332	29.00	5244549	562	18108	50.58
282	*Escherichia coli*	41.20	250	860844	20.00	4881325	1147	6818	50.62
296	*Escherichia coli*	6.95	250	683028	14.00	4404967	2679	2126	50.90
302	*Escherichia coli*	7.81	250	584544	13.00	4122924	2687	1927	50.90
316	*Escherichia coli*	9.24	250	726080	19.00	4492818	1754	3895	50.85
317	*Escherichia coli*	5.71	250	360166	7.00	2570598	2709	1002	51.65
325	*Escherichia coli*	7.53	250	546628	13.00	4147231	2629	2007	51.18
329	*Escherichia coli*	15.32	250	618314	10.00	5100491	3926	1609	54.59
334	*Escherichia coli*	6.83	250	2038924	19.00	6486059	2727	3536	52.31
340	*Escherichia coli*	47.80	250	579420	10.00	2905035	2897	1065	50.08
343	*Escherichia coli*	7.16	250	762358	18.00	4402662	1836	3422	50.60
347	*Escherichia coli*	8.75	250	839458	16.00	4054696	2739	1820	50.73
352	*Escherichia coli*	6.88	250	784682	17.00	4560681	2240	2724	50.78
359	*Escherichia coli*	10.97	250	675456	17.00	4221864	2168	2658	50.86
360	*Escherichia coli*	8.21	250	609908	14.00	4147515	2628	1999	50.94
364	*Escherichia coli*	7.42	250	762848	14.00	4009172	3127	1498	50.33
365	*Escherichia coli*	16.03	250	812870	16.00	3214283	3001	1188	50.18
1	*Enterococcus* spp.	5.58	250	2916736	62.00	2801553	128	39107	37.42
2	*Enterococcus* spp.	8.01	250	2050564	67.00	2784784	34	133639	37.52
11	*Enterococcus* spp.	8.29	250	2570334	72.00	2909675	33	164435	37.47

13	*Enterococcus* spp.	10.95	250	3421342	68.00	2888828	40	150611	37.38
16	*Enterococcus* spp.	9.07	250	3536538	70.00	2850398	108	43916	37.50
25	*Enterococcus* spp.	7.71	250	4080114	71.00	2902410	57	114073	37.35
26	*Enterococcus* spp.	12.44	250	3330112	71.00	2867878	53	125804	37.39
28	*Enterococcus* spp.	7.14	250	3506614	69.00	2835982	74	72041	37.43
35	*Enterococcus* spp.	6.97	250	3759442	73.00	2850038	85	79393	37.50
37	*Enterococcus* spp.	9.57	250	3627620	77.00	2840367	43	337002	37.40
40	*Enterococcus* spp.	8.36	250	4107948	74.00	2894765	67	79373	37.44
43	*Enterococcus* spp.	7.25	250	3447688	77.00	2810090	31	262969	37.46
52	*Enterococcus* spp.	15.12	250	3378284	68.00	2825460	137	39512	37.48
56	*Enterococcus* spp.	6.69	250	2953004	40.00	2438990	1148	2986	32.53
58	*Enterococcus* spp.	8.22	250	1310626	47.00	2675277	213	18943	37.60
61	*Enterococcus* spp.	10.21	250	1036470	33.00	2806542	323	14847	37.42
67	*Enterococcus* spp.	11.02	250	765210	10.00	4020255	3621	1173	46.03
76	*Enterococcus* spp.	6.88	250	757662	28.00	2808098	71	88075	37.42
86	*Enterococcus* spp.	8.00	250	1524000	39.00	2847762	304	15108	37.49
91	*Enterococcus* spp.	12.40	250	1207526	33.00	2835422	422	10563	37.46
94	*Enterococcus* spp.	20.21	250	787150	28.00	2717606	455	9857	37.39
100	*Enterococcus* spp.	5.62	250	1215690	24.00	2684785	779	5044	37.53
101	*Enterococcus* spp.	7.83	250	1089512	32.00	2716375	410	11695	37.54
103	*Enterococcus* spp.	14.10	250	1287280	30.00	2626461	451	9094	37.61
106	*Enterococcus* spp.	7.45	250	1305424	36.00	2808471	394	12246	37.40
113	*Enterococcus* spp.	8.72	250	1020628	29.00	2936207	335	15748	37.30
121	*Enterococcus* spp.	6.86	250	621792	21.00	2890702	144	44080	37.36
124	*Enterococcus* spp.	10.69	250	710788	17.00	2132021	1381	1905	32.51
130	*Enterococcus* spp.	8.34	250	1542974	43.00	2978422	286	17987	37.12
133	*Enterococcus* spp.	7.12	250	1333724	43.00	2849686	175	27673	37.49
136	*Enterococcus* spp.	16.96	250	1475206	41.00	2861106	290	16001	37.45
140	*Enterococcus* spp.	6.77	250	756386	19.00	2641213	345	12595	37.62
142	*Enterococcus* spp.	7.91	250	630040	17.00	2807323	491	10381	37.44
145	*Enterococcus* spp.	12.49	250	431746	11.00	2411363	1412	2269	37.51
152	*Enterococcus* spp.	7.22	250	1657078	40.00	2820421	197	24397	37.44
154	*Enterococcus* spp.	8.05	250	1705780	38.00	2855291	311	15097	37.46
157	*Enterococcus* spp.	5.94	250	1937790	43.00	2859241	293	16380	37.45
160	*Enterococcus* spp.	7.55	250	2131840	45.00	2760385	268	15970	37.53

163	*Enterococcus* spp.	13.39	250	2359796	37.00	2909616	387	11469	37.25
164	*Enterococcus* spp.	7.28	250	1332912	29.00	2827678	283	16662	37.59
167	*Enterococcus* spp.	9.15	250	2092968	51.00	2888190	102	59752	37.36
169	*Enterococcus* spp.	6.66	250	2380952	34.00	3163781	600	8522	36.89
184	*Enterococcus* spp.	10.79	250	255800	7.00	662990	870	730	34.22
196	*Enterococcus* spp.	8.17	250	2030490	39.00	2853325	399	11727	37.47
197	*Enterococcus* spp.	7.37	250	1960628	53.00	2745290	81	53349	37.50
203	*Enterococcus* spp.	14.64	250	1766156	32.00	2929746	438	10825	37.24
208	*Enterococcus* spp.	6.80	250	2136022	35.00	2978520	484	10031	36.91
211	*Enterococcus* spp.	7.63	250	2029966	58.00	2840075	168	33164	37.49
214	*Enterococcus* spp.	11.31	250	2003782	41.00	2690861	418	11321	37.56
217	*Enterococcus* spp.	7.10	250	2169046	38.00	2660658	720	5375	37.54
223	*Enterococcus* spp.	8.89	250	392060	9.00	485507	653	695	37.05
224	*Enterococcus* spp.	5.74	250	1738306	32.00	2664351	577	7448	37.68
250	*Enterococcus* spp.	7.87	250	1327182	18.00	2530222	1216	2796	38.15
260	*Enterococcus* spp.	12.46	250	2080540	52.00	2880959	163	32922	37.44
266	*Enterococcus* spp.	7.20	250	1684216	61.00	2894794	81	82646	37.45
268	*Enterococcus* spp.	4.38	250	1690162	21.00	4977290	3080	2052	34.28
274	*Enterococcus* spp.	5.14	250	688502	17.00	2705759	967	3797	37.33
277	*Enterococcus* spp.	7.52	250	1933220	64.00	2714324	157	31131	37.56
278	*Enterococcus* spp.	10.23	250	1418410	53.00	2793867	65	79406	37.59
283	*Enterococcus* spp.	6.91	250	1506592	38.00	3002650	698	7210	39.85
286	*Enterococcus* spp.	8.76	250	1190412	39.00	2700683	140	30633	37.60
298	*Enterococcus* spp.	13.11	250	581886	23.00	2143330	1241	2248	38.15
299	*Enterococcus* spp.	7.33	250	1646564	51.00	3176599	105	52536	37.01
316	*Enterococcus* spp.	18.58	250	2063540	68.00	2880397	80	54764	37.48
319	*Enterococcus* spp.	10.30	250	1401312	48.00	2790896	152	29468	37.49
320	*Enterococcus* spp.	11.60	250	1676230	58.00	2793179	117	45307	37.50
328	*Enterococcus* spp.	17.60	250	1796282	60.00	2881648	114	46320	37.47
329	*Enterococcus* spp.	18.90	250	1506264	50.00	2877141	131	46080	37.46
331	*Enterococcus* spp.	14.80	250	1835936	63.00	2828300	81	78936	37.45
332	*Enterococcus* spp.	16.30	250	1157424	38.00	2744351	166	30034	37.50
334	*Enterococcus* spp.	20.70	250	1849112	71.00	2823021	31	267510	37.45
335	*Enterococcus* spp.	12.50	250	1734212	58.00	2816295	119	42085	37.45
340	*Enterococcus* spp.	22.80	250	1745428	62.00	2806094	79	75419	37.40

343	*Enterococcus* spp.	17.30	250	2210032	70.00	2940202	132	57242	37.34
350	*Enterococcus* spp.	19.70	250	1726570	50.00	3009116	261	18371	37.09
352	*Enterococcus* spp.	15.20	250	1661252	51.00	3181303	98	72031	37.00
356	*Enterococcus* spp.	14.90	250	1266650	47.00	2692658	78	57232	37.65
362	*Enterococcus* spp.	2.55	250	1350900	31.00	4129081	439	15634	39.96
365	*Enterococcus* spp.	6.38	250	1200872	37.00	2746839	302	14822	37.48
367	*Enterococcus* spp.	22.80	250	2146734	67.00	2878837	120	38650	37.66
373	*Enterococcus* spp.	4.69	250	1718894	50.00	2810106	294	17947	32.35
376	*Enterococcus* spp.	14.00	250	1434784	50.00	2726911	130	36187	37.65
377	*Enterococcus* spp.	16.00	250	1605392	55.00	2885825	124	46587	37.46
379	*Enterococcus* spp.	13.50	250	2211812	58.00	3068840	169	38862	36.58
382	*Enterococcus* spp.	14.30	250	1241660	22.00	4636935	1748	4116	37.81
388	*Enterococcus* spp.	14.50	250	2091432	67.00	2949035	128	92844	37.55
394	*Enterococcus* spp.	13.80	250	1889746	73.00	2686093	56	102360	37.62
397	*Enterococcus* spp.	19.00	250	1736066	65.00	2826641	25	278626	37.59
400	*Enterococcus* spp.	19.70	250	1770992	72.00	2691938	27	206962	37.63
407	*Enterococcus* spp.	14.20	250	1819084	63.00	2835704	102	55690	37.33
409	*Enterococcus* spp.	23.20	250	1734688	66.00	2724120	34	178031	37.60
412	*Enterococcus* spp.	20.60	250	1571000	50.00	2976190	92	57248	37.33
416	*Enterococcus* spp.	18.90	250	1362906	49.00	2888204	114	50920	37.45
418	*Enterococcus* spp.	17.70	250	1898074	58.00	3141014	953	5379	37.22
419	*Enterococcus* spp.	12.30	250	956254	33.00	2764707	177	25909	37.58
421	*Enterococcus* spp.	21.40	250	1712286	65.00	2688827	37	111202	37.65
424	*Enterococcus* spp.	2.75	250	1305012	32.00	3334944	742	10335	39.43
427	*Enterococcus* spp.	16.00	250	1350506	51.00	2884254	41	179982	37.43
428	*Enterococcus* spp.	5.31	250	1532806	47.00	2814868	246	19839	37.59
431	*Enterococcus* spp.	9.67	250	1233276	39.00	2959543	189	26893	37.25
433	*Enterococcus* spp.	16.20	250	1653674	54.00	3041202	81	83207	36.89
442	*Enterococcus* spp.	18.00	250	1811864	70.00	2772532	45	123730	37.52
445	*Enterococcus* spp.	26.10	250	1650760	62.00	2733218	49	98570	37.54
451	*Enterococcus* spp.	18.70	250	1611826	59.00	2700434	75	74859	37.57
454	*Enterococcus* spp.	20.00	250	611002	21.00	2779790	203	22902	37.42
466	*Enterococcus* spp.	3.63	250	1197832	26.00	4604848	363	24450	39.76
472	*Enterococcus* spp.	19.40	250	1909798	62.00	3003836	97	52546	37.16
473	*Enterococcus* spp.	2.10	250	1208890	31.00	3008219	440	11108	36.56

478	*Enterococcus* spp.	7.49	250	606190	16.00	2888459	728	6024	36.55
481	*Enterococcus* spp.	3.86	250	623376	10.00	1561298	1593	1042	35.55
484	*Enterococcus* spp.	7.28	250	1529852	37.00	3585438	688	13506	37.66
2	*Salmonella* spp.	8.52	250	2830490	60.00	5010873	56	190442	52.04
5	*Salmonella* spp.	8.41	250	3363878	67.00	5051726	61	158297	52.01
7	*Salmonella* spp.	32.50	250	1363296	24.00	5514901	812	60468	52.49
10	*Salmonella* spp.	45.70	250	1061948	22.00	4940634	166	120381	53.65
20	*Salmonella* spp.	6.73	250	2791616	58.00	4809901	63	156951	52.19
24	*Salmonella* spp.	38.90	250	1084668	22.00	4904463	162	75052	53.78
34	*Salmonella* spp.	7.06	250	3137528	64.00	4784469	76	130197	52.19
37	*Salmonella* spp.	12.15	250	3742856	76.00	4834390	52	196636	52.18
40	*Salmonella* spp.	6.90	250	3224092	67.00	4842521	81	144180	52.15
49	*Salmonella* spp.	7.59	250	2964906	64.00	4892464	67	196635	52.16
61	*Salmonella* spp.	30.30	250	1005156	20.00	4843366	331	34418	52.10
72	*Salmonella* spp.	32.60	250	1188650	22.00	5653454	780	110381	53.53
79	*Salmonella* spp.	35.80	250	1109486	23.00	4967456	181	113933	53.74
82	*Salmonella* spp.	29.50	250	1205098	27.00	4842925	98	126196	53.64
83	*Salmonella* spp.	11.30	250	2159492	37.00	4739632	291	29581	52.02
85	*Salmonella* spp.	9.21	250	2981454	53.00	4756688	168	53275	52.01
87	*Salmonella* spp.	34.00	250	1181908	14.00	8698124	2812	6328	52.98
93	*Salmonella* spp.	22.50	250	823950	16.00	4872550	310	30381	53.78
109	*Salmonella* spp.	32.70	250	1307424	18.00	6809417	1981	7174	53.15
115	*Salmonella* spp.	7.86	250	2150482	52.00	3873132	72	160869	38.49
119	*Salmonella* spp.	35.00	250	974446	12.00	7917861	3700	3790	52.69
120	*Salmonella* spp.	11.08	250	2585926	51.00	4860210	110	107231	52.12
136	*Salmonella* spp.	7.39	250	2303618	37.00	4849813	244	35635	52.00
139	*Salmonella* spp.	8.61	250	2280436	36.00	4836912	303	29589	51.99
140	*Salmonella* spp.	31.40	250	1659374	19.00	9640049	374	47573	52.99
146	*Salmonella* spp.	48.60	250	919700	16.00	5527900	1059	25674	52.35
157	*Salmonella* spp.	35.90	250	1219970	23.00	5429382	657	52216	52.25
168	*Salmonella* spp.	5.81	250	1909118	34.00	4850140	175	50035	52.17
177	*Salmonella* spp.	31.70	250	1072706	23.00	4873733	118	83634	53.80
178	*Salmonella* spp.	7.66	250	2071538	39.00	4799618	138	67119	52.17
187	*Salmonella* spp.	13.27	250	2371076	31.00	4698859	573	13435	52.16
207	*Salmonella* spp.	7.18	250	1753124	27.00	4729657	339	26058	52.20

209	*Salmonella* spp.	9.00	250	2106902	36.00	4745914	204	43746	52.20
260	*Salmonella* spp.	10.94	250	2035178	30.00	4716446	478	17217	52.15
